# Supplementary figures and images for: Escherichia coli adaptation under prolonged resource exhaustion is characterized by extreme parallelism and frequent historical contingency
Source: PLoS Genet. 2024 Jun 17;20(6):e1011333. doi: 10.1371/journal.pgen.1011333 (PMC11213340; doi:10.1371/journal.pgen.1011333)

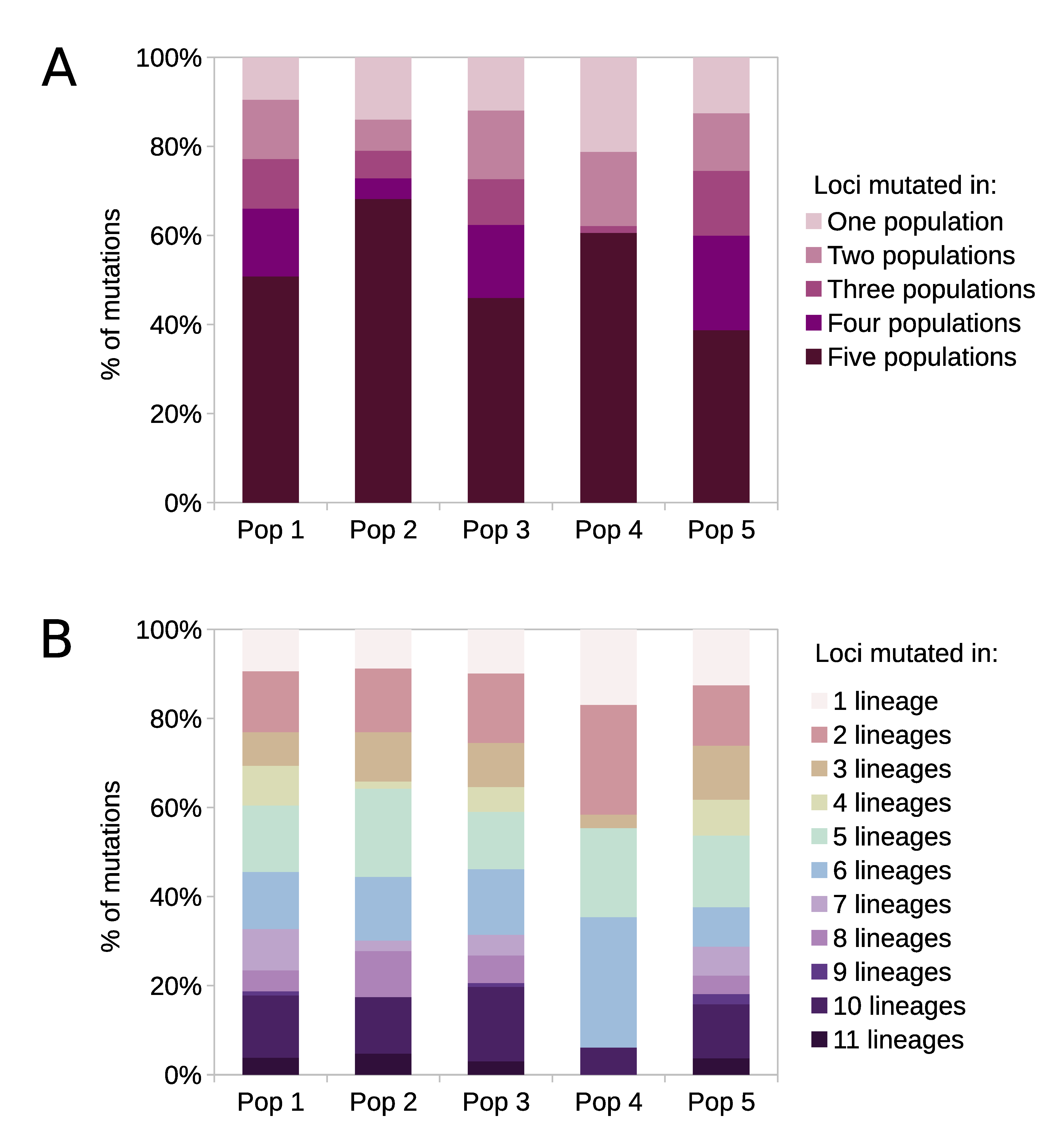

Supplement: S1 Fig — The charts show the fractions of mutations occurring in non-mutators (A) across populations; (B) across lineages; (TIF) [file pgen.1011333.s012.tif]

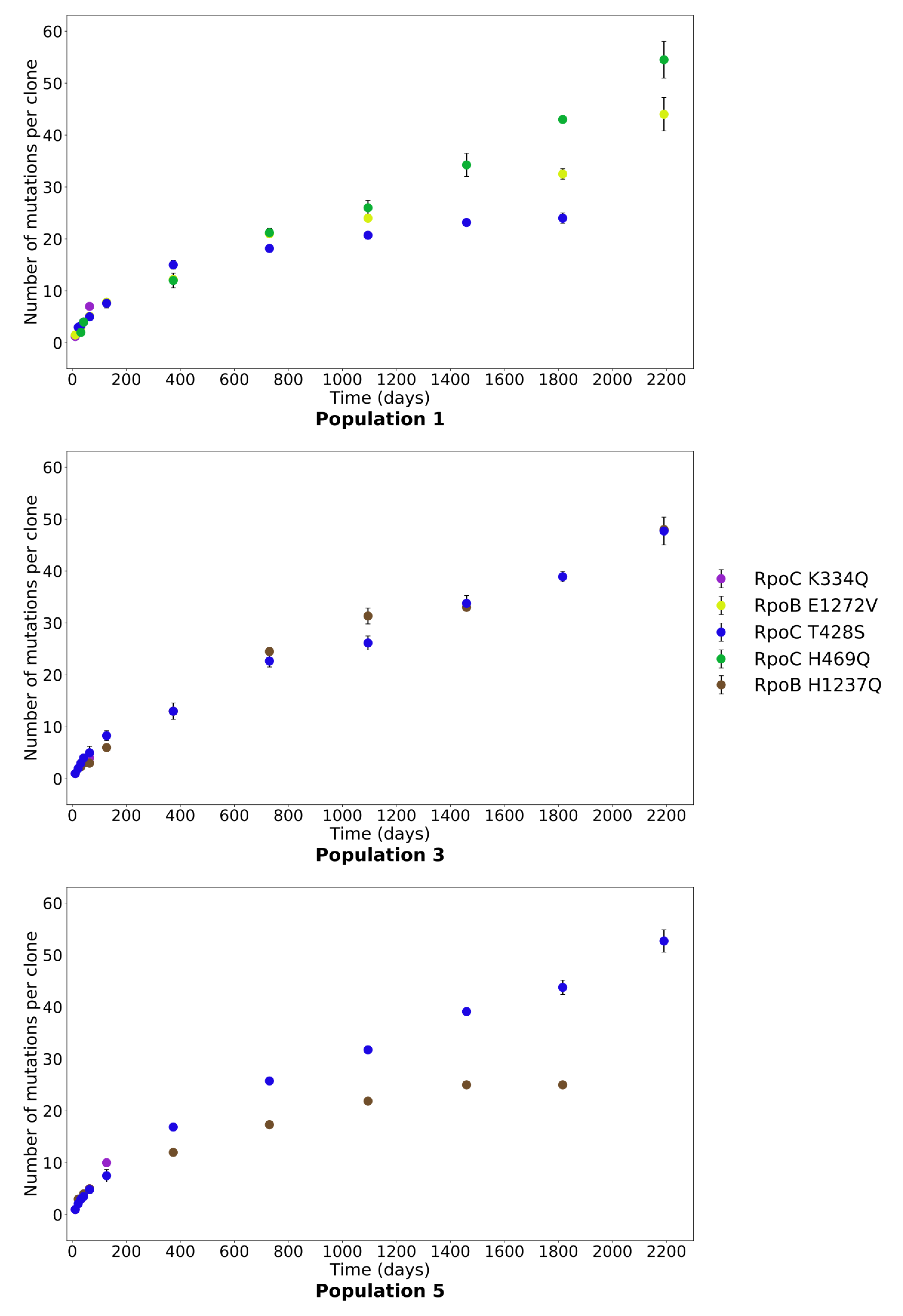

Supplement: S2 Fig — Data from the RpoB E1272G mutator lineages are not presented. Dots represent mean values across clones within each lineage with error bars representing standard deviations around these means. (TIF) [file pgen.1011333.s013.tif]
